# Characteristics of Phase IV Clinical Trials in Oncology: An Analysis Using the ClinicalTrials.gov Registry Data

**DOI:** 10.3390/curroncol30060443

**Published:** 2023-06-20

**Authors:** Brandon Michael Henry, Giuseppe Lippi, Ameen Nasser, Patryk Ostrowski

**Affiliations:** 1 Cmed Research Inc., Morrisville, NC 27560, USA; 2 Clinical Laboratory, Division of Nephrology and Hypertension, Cincinnati Children’s Hospital Medical Center, Cincinnati, OH 45229, USA; 3 Youthoria, Youth Research Organization, 30-363 Kraków, Poland; patryk.ostrowski@student.uj.edu.pl; 4 Section of Clinical Biochemistry and School of Medicine, University Hospital of Verona, 37129 Verona, Italy; giuseppe.lippi@univr.it; 5 Faculty of Medicine, Jagiellonian University Medical College, 31-008 Kraków, Poland; ameen.nasser@student.uj.edu.pl

**Keywords:** postmarketing surveillance, pharmacovigilance, registry data

## Abstract

The present study analyzed the characteristics of phase IV clinical trials in oncology using data from the ClinicalTrials.gov registry. The included trials were conducted between January 2013 and December 2022 and were examined for key characteristics, including outcome measures, interventions, sample sizes, and study design, different cancer types, and geographic regions. The analysis included 368 phase IV oncology studies. An amount of 50% of these studies examined both safety and efficacy, while 43.5% only reported efficacy outcome measures, and 6.5% only described safety outcome measures. Only 16.9% of studies were powered to detect adverse events with a frequency of 1 in 100. Targeted therapies accounted for the majority of included studies (53.5%), with breast (32.91%) and hematological cancers (25.82%) being the most frequently investigated malignancies. Most phase IV oncology studies lacked sufficient power to detect rare adverse events due to their small sample sizes and instead focused on effectiveness. To ensure that there is no gap in drug safety data collection and detection of rare adverse events due to limited phase IV clinical trials, there is a significant need for additional education and participation by both health care providers and patients in spontaneous reporting processes.

## 1. Introduction

Cancer is a complex and devastating illness that impacts millions of lives globally every year. Despite significant advances in cancer treatment, it remains the second leading cause of death worldwide [1]. Phase IV clinical trials in oncology are conducted subsequent to a cancer medication or treatment being cleared for market release. The general objective of these trials is to monitor the safety and efficacy/effectiveness of the cancer treatment among a larger population and for an extended duration. Such trials are often more open and entail the accumulation of data from individuals often enrolled under less rigid eligibility criteria than phase III trials, with participation guided by the permissible indications and contra-indications of the drug in accordance with the package insert [2]. Importantly, such studies aim to identify any latent and rarer safety concerns and focus on effectiveness, as opposed to strictly drug efficacy, characterized by less intensive monitoring and compliance, in line with real-world use of a drug/device in practice [2].

Post-marketing surveillance (PMS) is the practice where marketed medications are monitored for potential adverse events (AEs) post phase III clinical trials and may consist of active (randomized or observational phase IV studies, large data review) or passive (spontaneous reporting) processes [3]. PMS studies may help to evaluate the safety and effectiveness of novel therapeutics more precisely and extensively than clinical trials, especially with respect to rare AEs [4]. The safety profile of a novel therapeutic may not be able to be adequately investigated by the phase I-III trials due to various limitations, including small sample sizes, strict inclusion/exclusion criteria, less diverse populations, and their short duration [5]. As such, rarer AEs or those occurring only in specific populations may often be missed or go unnoticed in earlier phases of development. In some situations, such studies may be mandated by regulatory agencies. 

Importantly, roughly 20% of drugs received additional black box warnings during the post marketing phase, while 4% of the drugs were eventually withdrawn due to safety concerns [5]. PMS studies in oncology may provide more realistic results regarding the safety and effectiveness of novel cancer therapeutics, as they take place in a more natural setting [6]. However, there is a lack of information regarding the general characteristics, design, and attributes of phase IV clinical trials. 

ClinicalTrials.gov is a database provided by the U.S. National Library of Medicine on behalf of the National Institutes of Health (NIH), which stores data concerning both privately and publicly funded clinical studies conducted around the world. It was first launched in February 2000 [7], and, starting in 2005, the International Committee of Medical Journal Editors made it mandatory for clinical trials to be registered before publication [8]. Furthermore, under the Food and Drug Administration Amendments Act of 2007, sponsors or their representatives must register trials and report important data elements and basic trial results at ClinicalTrials.gov since 2007 [9]. Therefore, the ClinicalTrials.gov database is widely regarded as the most comprehensive global source of clinical trial information [5].

In the present investigation, the general characteristics of registered phase IV oncology clinical trials in oncology were explored by analyzing studies on ClinicalTrials.gov that were initiated over the last decade (2013–2022). The analysis included a thorough investigation of the various types of interventions examined by the phase IV clinical trials, the indications, their outcome measures, funding, study design, and more. 

## 2. Materials and Methods

### 2.1. Design and Data Source

In this retrospective observational study, the characteristics of phase IV clinical trials in oncology that were registered with ClinicalTrials.gov with a start date during the timeframe of 1 January 2013, to 31 December 2022 were investigated. To be eligible for inclusion in this analysis, a registered trial must be interventional and evaluate an anti-cancer drug or device or combination of drugs or drug/device in a defined population of cancer patients. Drugs without an anti-tumor effect, such as anesthetics or supportive therapies (ex., drugs for cytopenia), behavioral, diagnostic, prevention, or public health studies, were excluded. To facilitate the present analysis, a dataset of phase IV clinical studies from ClinicalTrials.gov, which was downloaded following the search and selection process described below and secured from the website on 31 March 2023, was obtained. Additionally, the authors of the present study designed a database to assist in the current analysis, analogically to previous investigations of ClinicalTrials.gov [5,10,11]. 

### 2.2. Study Selection

**Figure 1:**
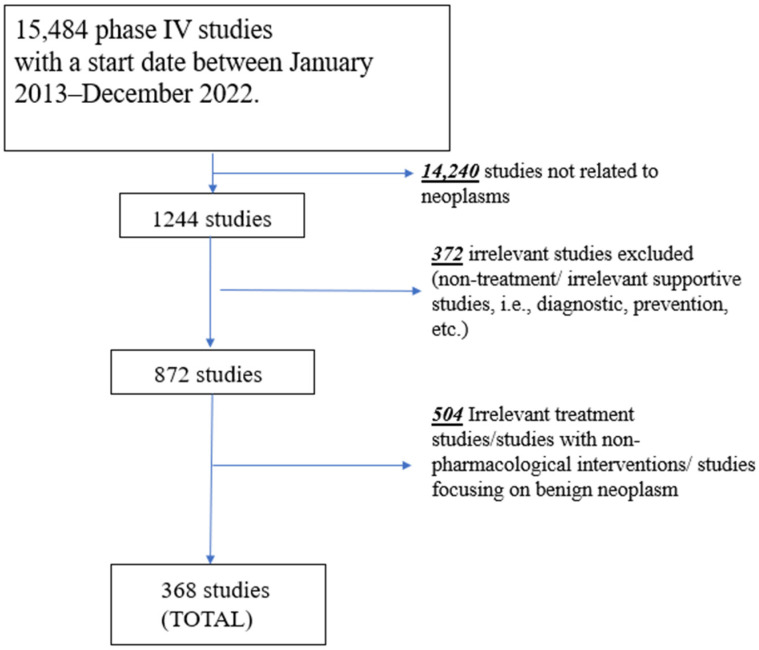
Flow chart of study selection and eligibility assessment.

An initial search of ClinicalTrials.gov for phase IV studies was conducted with a start date between 1 January 2013, to 31 December 2022, registered by 31 March 2023. The study selection process is presented in Figure 1. A total of 15,484 phase IV studies were initially identified. Following the use of a filter term, “neoplasms” (which includes terms “cancer” and “tumor”), in line with the terminology of the registry topic selection filter, 14,240 studies were excluded, leaving 1244 potentially eligible studies. Next, studies were excluded by their primary purpose, which can be found in the “study design” section in ClinicalTrials.gov. All non-treatment studies (i.e., prevention, diagnostic, supportive care, health services, and basic science) were excluded (*n* = 372). Next, the remaining 872 studies were reviewed independently by two authors (PO, AN) to exclude all studies focusing on non-oncological pathologies (i.e., rheumatoid arthritis, etc.) that were not excluded using the above filters and studies focused on broad cohorts with various indications (not specific to cancer patients). Moreover, if studies were not exclusive to or strictly focused on therapeutic agents (defined as drugs or devices) as a treatment for cancer or were non-pharmacologic studies (surgery or radiotherapy), they were excluded as per the a priori eligibility criteria (*n* = 504). After the eligibility review process, a total of 368 studies remained eligible and were included in this investigation. A list of the included studies with their identification number and title was added as supplementary material.

### 2.3. Data Collection

The ClinicalTrials.gov registry requires the sponsors or investigators of a clinical trial to report key trial data, which should include a set of data elements describing the study’s conditions, study design, sponsor, location, enrollment, eligibility criteria, and other protocol information. These data, which describe the general characteristics of the included studies, were extracted and analyzed. The said data included dates of the study, study design, outcome measures, cancer indication, age, sex, size of the cohorts, funding, location, and use of a data monitoring committee (DMC). 

The extracted characteristics of the clinical trials were later divided into more specific categories by a team of authors (PO, AN), with selected records checked for agreement by a third author (BMH). The outcome measures were divided into three categories: (1) safety, (2) efficacy, and (3) efficacy and safety, in accordance with the outcome measures recorded in the registry record. Due to limited data available, the authors of the present study did not attempt to distinguish between efficacy and effectiveness, thus using the term efficacy henceforth. The interventions were classified by type of anti-cancer therapeutics: (1), chemotherapy, which included alkylating agents, antimetabolites, antimicrotubule agents (mitotic inhibitors), topoisomerase inhibitors, and miscellaneous antineoplastics, (2) immunotherapy, which included cytokines, vaccine therapies, immunomodulatory drugs, and differentiating agents, (3) hormonal therapy, which consisted of antiestrogens, antiandrogens, aromatase inhibitors, luteinizing hormone-releasing hormone agonists, androgen biosynthesis inhibitors, androgens, corticosteroids, somatostatin analogs, and more, (4) targeted therapy, which included monoclonal antibodies, kinase inhibitors (targeting anaplastic lymphoma kinase, BRAF serine-threonine kinase, amongst others), proteasome inhibitors, mammalian target of rapamycin (MTORS), and more, (5) other anti-cancer therapy, (6) herbal/traditional medicine, (7) devices, and (8) other therapies. The indication for treatment (i.e., type(s) of cancers) was also extracted and analyzed according to system organ class. The included studies were also classified into sample size groups by total number of participants: (1) <300, (2) 300–599, (3) 600–2999, (4) ≥ 3000, and (5) missing. Same-size groups were determined based on the fact that, per binomial and Poisson distributions, enrollment must be greater than 300, 600, or 3000 to have a 95% chance of observing at least one AE with a probability of occurrence of 1%, 0.5%, or 0.1%, respectively (Table 1) [5,10]. 

In cases where a data field was incomplete (such as in the case of missing data concerning the allocation of the trial), we conducted a manual search (ClinicalTrials.gov) to attempt to retrieve the missing information. If the information was still unavailable on the website, we labeled the field as NA (not applicable) or missing. If a study presented an interventional model involving a single group and did not provide information on allocation and blinding, we made the assumption that allocation was non-randomized, and blinding was open. Nonetheless, if the single-arm studies were registered as randomized or blind, allocation or blinding was reported as “Missing/Uncertain/N.A” [5].

### 2.4. Statistical Analysis

Results were analyzed using descriptive statistics, with categorical data reported as absolute number (n) and relative frequency (%) and continuous variables reported as the median and interquartile range (IQR). Differences in frequencies of a variable between studies grouped by outcome measures were compared using the Chi-square test. Statistical analyses were carried out with Prism 8 (GraphPad Software, San Diego, CA, USA) with statistical significance set at *p* < 0.05.

This study was conducted in compliance with the Declaration of Helsinki and within the terms of local legislation. No ethical committee approval was required for performing this retrospective analysis of publicly available clinical trial registry data. 

## 3. Results

**Figure 2:**
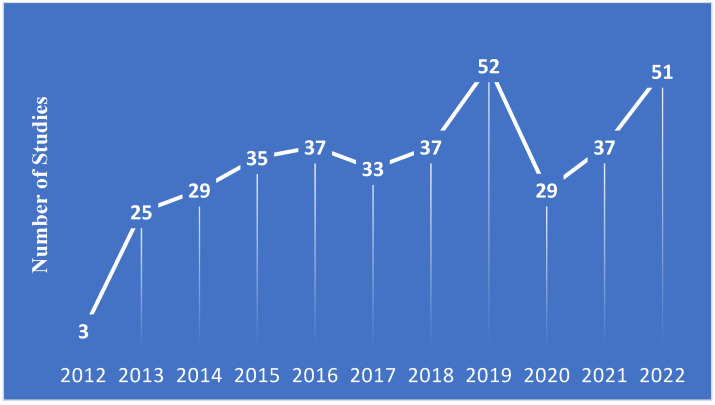
Line graph demonstrating the number of new studies started by year.

A total of 368 phase IV oncology studies started between January 2013 and December 2022 were included in the present analysis, 35.9% of which were currently reported as active as of 31 March 2023. Figure 2 shows the number of studies in the registry by start year. The full characteristics of the included studies are presented in Table 2. Among the included studies, only 19.8% were limited to a single-sex. The majority of studies (86.1%) did not include children (<18 years), while only 6.8% excluded elderly (>65 years) participants. Most of the included phase IV studies were focused on solid tumors (71.2%). 

The sample size of included studies ranged from 0 to 5000. The majority of included studies were small in size, with a median of 90 (IQR: 40–200), with 83.2% of studies consisting of <300 participants. Only one study (0.3%) was powered to detect AEs occurring at a rate of 0.1%, while only 21 (5.7%) and 32 (8.7%) studies were powered to detect AEs occurring at a rate of 0.5% and 1%, respectively. 

With respect to trial design, single-group assignment accounted for 50.0% of studies, while parallel assignment accounted for 47.0% of studies. All but 8.7% of included studies were open-label. The majority of studies were funded by industry (37.3%), followed closely by universities (or a similar institution) (34.5%) and hospitals (or a similar institution) (30.7%). Nearly half of all studies employed a Data Monitoring Committee (DMC; 49.5%). Over one-third of studies (39.4%) had started before submission and registration on ClincialTrials.gov. 

Exploring geographical distribution, a large majority of studies were conducted in Asia and Pacific regions (52.72%), which participated in more of the included studies than the next two largest regions, North America (17.9%) and Europe (17.1%) combined. Limited studies were performed in Africa (1.6%), Central and South America (3.3%), and the Middle East (1.4%). 

Looking at the reported outcome measures, exactly half of the studies looked at both safety and efficacy, whilst 43.5% reported only efficacy outcome measures, and 6.5% reported only safety outcome measures. The majority of safety-only studies were small in size, with 75% having <300 participants, and, thus, they were not powerful enough to detect AEs occurring at a rate of 1 in 100 participants with 95% confidence. Safety studies were not found to frequently have larger sample sizes (i.e., >300 participants) compared to efficacy-only studies (25.0% vs. 13.4%; *p* = 0.178). No statistically significant differences were observed between safety-only vs. efficacy-only studies (*p* > 0.05 for all comparisons, data not shown). 

Table 3 shows the characteristics of studies by type of pharmacologic therapy. Targeted therapies (53.5%) accounted for the majority of included studies, followed by chemotherapy (43.0%), hormonal therapies (17.7%), and immunotherapies (6.0%). Only fourteen (3.8%) studies utilized herbal/traditional medications, and two (0.5%) studies utilized devices. Key study characteristics were similar between types of pharmacologic therapy. Unsurprisingly, trials using hormonal therapies more frequently included only a single-sex (49.2%) compared to other types of interventions (0–20.3%). Of note, 14 (3.8%) trials combined a drug with surgical or radiotherapy intervention, while 64 (17.4%) trials were drug–drug combination studies. Interestingly, after the inclusion/exclusion process, none of the evaluated phase IV clinical trials analyzed CAT-T cells. However, numerous studies focused on various antibody-drug conjugates, such as brentuximab and trastuzumab. It is important to point out that no phase IV clinical trials analyzed the more recently approved antibody-drug conjugates, such as Loncastuximab or Belantamab.

**Figure 3:**
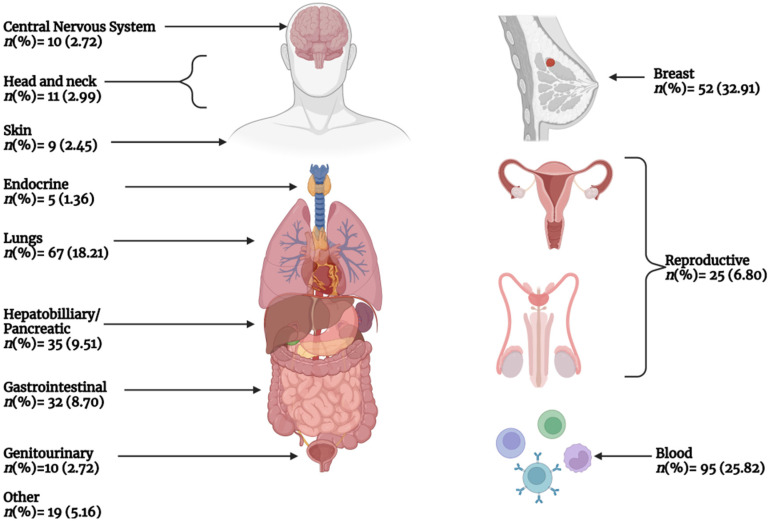
The number of phase IV studies started between 2013–2022 by primary cancer indication according to system organ class.

**Figure 4:**
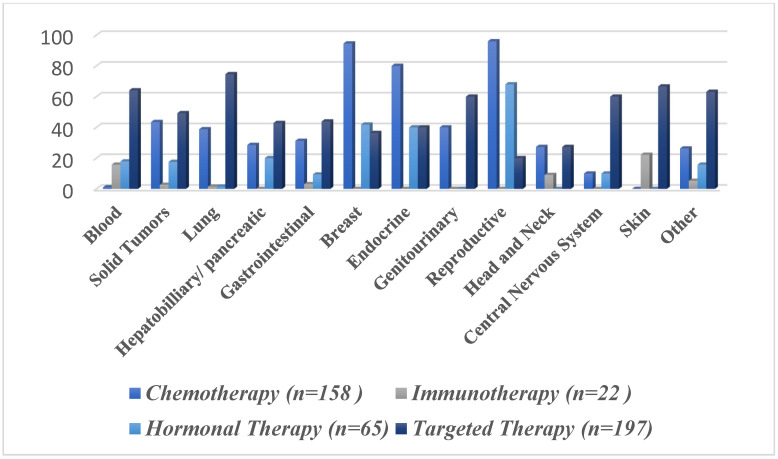
Frequency of different pharmacologic interventions by primary cancer indication according to system organ class. The “solid tumors” category consists of non-hematological malignancies, such as lung and breast cancers.

Figure 3 presents the number of included phase IV studies by primary cancer indication. Breast cancer accounted for nearly one-third (32.9%) of all studies, while hematologic malignancies accounted for slightly over a quarter (25.8%) of all studies, and lung cancer accounted for 18.21% of all studies. All other cancers accounted for <10% of all studies. Figure 4 demonstrated the frequency of different interventions by cancer indication, while the distribution of intervention by cancer type is presented in Table 4. 

## 4. Discussion

The present study aimed to demonstrate the characteristics of phase IV clinical trials in oncology over the last decade, using data obtained from the ClinicalTrials.gov registry. This analysis examined various aspects of the trials, including their outcome measures, interventions, sample sizes, and study design. The study also looked at the distribution of phase IV trials across different cancer types and geographic regions. By analyzing these factors, this study aimed to identify areas of strengths and weaknesses in the design and implementation of phase IV trials, with the ultimate goal of improving the quality and effectiveness of these trials in the future. The present analysis exhibits several noteworthy strengths that contribute to its overall quality and credibility. This study encompassed a detailed and informative evaluation of the existing phase IV clinical trials registered in the ClinicalTrials.gov database, specifically focusing on assessing the safety of various drugs. Moreover, a strict analysis process, widely employed for evaluating data from ClinicalTrials.gov, was followed in order to yield compelling results.

The present retrospective observational analysis included a total of 368 phase IV oncology studies. A recent analysis conducted by Zhang et al. [5] found 251 phase IV studies between 2004–2014 using similar parameters as the presented study (in congruence with recent advances in oncology, especially targeted therapies in the last decade). The rapid rise in new anti-cancer therapeutics, such as immunotherapies and targeted therapies, necessitates the need for more phase IV clinical trials to be conducted for proper post-market safety control [12]. Olivier et al. [12] conducted a cross-sectional study where they analyzed the approval rate of anticancer drugs based on their mechanism of action. Their analysis included a total of 332 approvals, and between 2009 and 2020, there was an increase in the total number of approvals from 8 to 57. Furthermore, according to their findings, when considering all tumor types combined, 16% of drug approvals were based on a novel mechanism of action. However, when looking at each individual tumor type separately, the percentage of drug approvals based on a new mechanism of action increased to 37%. However, the present analysis demonstrates that, despite the rapid rise in approved therapeutics, post-market safety remains dependent on passive processes, including spontaneous surveillance systems, such as FDA adverse event reporting system (FAERS). However, these spontaneous reporting programs, unfortunately, encounter numerous limitations in detecting AEs, including poor health, significant under-reporting, limited participation, incomplete reports, and bias (i.e., Weber Effect), and, thus, they are likely underutilized, hampering the full assessment of patient safety [6]. While some of this may be overcome in the future with utilization of big data approaches (such as analysis of electronic medical records), in order to fully address the problem of under-reporting, healthcare providers need to be educated and trained on pharmacovigilance and adverse drug reaction (ADR) reporting systems. Simple and accessible channels for reporting ADRs should be established, such as phone hotlines and user-friendly computer tools designed for reporting ADRs. This will encourage healthcare providers to report any ADRs they encounter during their busy work schedules. Pharmacovigilance centers can incentivize healthcare providers to report by recognizing their efforts, providing feedback on reported cases or pharmacovigilance activities, and offering support, such as clinical advice [13]. Moreover, further patient education and participation in the spontaneous reporting processes should be encouraged. Furthermore, encouraging sponsors and investigators of clinical trials to provide complete and accurate information in registration forms is crucial for ensuring transparency and scientific integrity in the research process. By emphasizing the importance of comprehensive reporting, it enables regulatory authorities, healthcare professionals, and the general public to make well-informed decisions regarding the trial’s methodology, outcomes, and safety. This transparency not only enhances trust in the research community, but it also facilitates the identification of potential biases or discrepancies, leading to more reliable and meaningful conclusions that can ultimately improve patient care and advance medical knowledge.

The most frequently analyzed oncological interventions in the last decade were therapeutics regarded as targeted therapy (53.5%), chemotherapy (42.9%), hormonal therapy (17.6%), and immunotherapy (5.9%). Moreover, the characteristics of the study design regarding these interventions were also investigated. Unsurprisingly, the majority of the aforementioned anti-cancer therapies were open-labeled trials (94.4%, 92.5%, 86.1%, and 95.4%, respectively) (Table 3). The intervention model varied considerably between these therapeutic interventions. The present study shows that the parallel arm design was the most frequently used approach in clinical trials investigating chemotherapeutic (59.4%) and immunotherapeutic agents (45.4%). However, the single-group assignment was the most commonly utilized design in both hormonal (50.7%) and targeted therapies (63.4%). The studies which investigated chemotherapeutic treatments used most commonly subject groups with breast (32.9%) or lung (16.4%) cancer. Moreover, the hormonal therapies were mostly investigated in cohorts with breast (35.3%), reproductive (26.1%), and blood cancers (26.1%). Targeted therapies and immunotherapies were most frequently investigated in patients with blood cancers (30.9% and 68.1%, respectively). 

The size of the sample used in a clinical trial is tightly related to the quality of safety surveillance of the investigated intervention [5]. Phase IV trials of a smaller scale can be employed to assess the efficacy of a specific medication within a particular subset of patients or in unique circumstances [5,14]. However, there are various concerns regarding phase IV clinical trials with smaller sample sizes, particularly due to the reduced safety surveillance of the investigated intervention. The majority of the studies analyzed in the present paper had a low sample size, i.e., ≤299 (83.1%). Unfortunately, the majority of the said studies still had a sample size below 300 subjects (75.0%). The results in the present analysis demonstrate that oncological phase IV clinical trials in the last decade have not been appropriate for safety surveillance, especially for detecting rare AEs.

Interestingly, the lead sponsor of the analyzed studies varied significantly. The majority of the studies were sponsored by either an industry-related sponsor (37.2%), a hospital (or a similar institution) (30.7%), or a university (or a similar institution) (34.5%). The presence of DMC was also investigated in the present study. The results of the current study show that the DMC was reported in 49.4% of the clinical trials. However, it was absent in almost one-third of studies (29.1%) and not reported or missing in 21.4%. It is clear that taking advantage of DMC, even in open-label clinical trials, can help to monitor their safety, as well as assess their overall risks and benefits. Having a DMC in phase IV clinical trials provides an independent assessment of the safety, scientific validity, and integrity of the studies. If the DMC is used, it should be independent from the study sponsor. This independence enables the DMC to provide credible and unbiased recommendations to the sponsors. It ensures that sponsors can make study decisions without being influenced by their knowledge of interim study results. Alongside monitoring trial safety, the DMC also plays a crucial role in evaluating the trial’s ongoing validity and scientific quality. They ensure that clinical equipoise is maintained throughout the trial and monitor essential aspects, such as participant recruitment, protocol compliance, and data quality [15]. The results of the present study show that a considerable amount of clinical trials (39.4%) were started before being submitted to ClinicalTrials.gov. This could be indicative of potential bias. ClinicalTrials.gov maintains a log of any changes to the registration of a study. Thus, changes in design or study characteristics can be tracked. If a study starts before registration, there is a potential for bias, as changes could have been implemented after the study started, but these would not be captured in the registry. Moreover, retrospective data collection is associated with a potential information bias due to missing data that may occur as a result of inadequate registration quality or the omission of variables that were not anticipated to be registered beforehand.

The present analysis demonstrates that the majority of the clinical trials were conducted in Asia and the Pacific region (52.7%). This was also demonstrated by other studies in the past [5]. However, a very limited number of clinical trials were conducted in Africa (1.6%) and the Middle East (1.3%). This is a major limitation in post-market surveillance studies, as we may not gather a sufficient understanding of how these drugs work in other diverse populations. Hence, it is incredibly important to include more countries from these regions in future studies. Furthermore, approximately 10% of the included clinical trials did not provide data concerning the region. This may limit the overall understanding of the global picture of the clinical phase IV trials. The overall status of the clinical trials included in the present analysis was also investigated. It was established that 25.2% of the studies were still recruiting subjects, and 20.1% were completed. Interestingly, 5.4% of the clinical trials were terminated, which is higher than what was reported in a previous study conducted by Califf et al. [10] (3.3%). However, this was lower than what the more recent analysis conducted by Zhang et al. [5] reported (8.6%). 

The present study has various limitations that need to be addressed. There is limited available data in the registry, and not all phase IV studies are registered. Hence, some ongoing clinical trials may not have been included in the present analysis. Furthermore, there is a high amount of missing/uncertain data; the sponsors/investigators should be further encouraged to fully complete all fields of the registration form. However, the authors of the present study made a manual screening of all the studied records; this helped address some missing data issues and improved the overall quality of the present report. Lastly, as mentioned earlier, almost 1/3 of studies started before being registered in ClinicalTrials.gov; this can create a bias in both the results of the said clinical trials, as well as the current analysis of study attributes. 

## 5. Conclusions

The present analysis demonstrated that targeted therapies (53.53%) account for the majority of all recent phase IV oncology studies, showing a rapid development of this field. Furthermore, the number of phase IV clinical trials has increased significantly over previous analyses. However, the clinical trials included in the present investigation were dominated by small sample sizes, with most of the studies enrolling less than 300 individuals (83.15%). Hence, most of the phase IV studies are not sufficiently powered to detect rare AEs and instead are focused on efficacy/effectiveness. Due to the limited number of ongoing safety studies, there is a significant requirement for additional education on and participation by both healthcare providers and patients in spontaneous reporting processes, thus ensuring that there is no gap in clinical safety data collection due to limited phase IV clinical trials. This would be especially true with respect to rare AEs, which may not have been identified in clinical development and which were only observed when the drug is utilized in a broader population in routine clinical practice. 

## Figures and Tables

**Table 1:** Patients needed to enroll to observe rare adverse events.

Anticipated Incidence of a Given Adverse Event (Participants)	Anticipated Incidence of a Given Adverse Event (Rate)	Number of Participants Needed to Observe at Least 1 Given Adverse Event
1 in 100	1%	300
1 in 200	0.5%	600
1 in 1000	0.1%	≥3000

**Table 2:** Characteristics of the included phase IV clinical trials by outcome measure *.

	Number (%)			
	All (*n* = 368)	Safety Alone (*n* = 24)	Efficacy Alone (*n* =160)	Safety/Efficacy (*n* =184)
Overall status				
Not yet recruiting	27 (7.33)	0 (0.00)	8 (5.00)	19 (10.33)
Recruiting	93 (25.27)	8 (33.33)	34 (21.25)	51 (27.72)
Completed	74 (20.10)	3 (1.25)	35 (21.88)	36 (19.57)
Suspended	1 (0.27)	0 (0.00)	1 (0.625)	0 (0.00)
Terminated	20 (5.43)	1 (4.17)	12 (7.50)	7 (3.80)
Withdrawn	8 (2.17)	0 (0.00)	2 (1.25)	6 (3.26)
Active, not recruiting	34 (9.24)	3 (12.50)	10 (6.25)	21 (11.41)
Enrolling by invitation	5 (1.36)	0 (0.00)	4 (2.50)	1 (0.54)
Unknown Status	106 (28.80)	9 (37.5)	54 (33.75)	43 (23.37)
Intervention				
Chemotherapy	158 (42.93)	9 (37.5)	71 (44.38)	78 (42.39)
Immunotherapy	22 (5.99)	0 (0.00)	14 (8.75)	8 (4.35)
Hormonal Therapy	65 (17.66)	3 (12.5)	32 (20.00)	1 (1.63)
Targeted Therapy	197 (53.53)	14 (58.33)	82 (51.25)	101 (54.89)
Herbal/Traditional Medicine	14 (3.80)	1 (4.17)	6 (3.75)	7 (3.80)
Device	2 (0.54)	0 (0.00)	1 (0.63)	1 (0.54)
Other	36 (9.78)	1 (4.17)	20 (12.5)	15 (8.152)
Type of Cancer				
Solid Tumor	262 (71.20)	23 (95.83)	104 (65.00)	135 (73.37)
Hematologic Malignancy	95 (25.82)	1 (4.12)	53 (33.13)	41 (22.28)
Not Specified	11 (2.99)	0 (0.00)	3 (1.88)	8 (5.63)
Enrollment				
1–299	306 (83.15)	18 (75.00)	138 (86.25)	146 (79.35)
300–599	32 (8.70)	3 (12.50)	11 (6.88)	19 (10.33)
600–2999	21 (5.71)	3 (12.50)	12 (6.52)	12 (6.52)
≥3000	1 (0.27)	0 (0.00)	0 (0.00)	1 (0.54)
Intervention model				
Crossover assignment	4 (1.10)	1 (4.17)	2 (1.25)	1 (0.54)
Single group assignment	184 (50.00)	14 (58.33)	82 (51.25)	88 (47.83)
Parallel assignment	173 (47.01)	9 (37.50)	72 (45.00)	92 (50.00)
Factorial assignment	0 (0.00)	0 (0.00)	0 (0.00)	0 (0.00)
Missing	7 (1.90)	4 (2.50)	4 (2.50)	3 (1.63)
Allocation				
Randomised	153 (41.58)	10 (41.67)	62 (38.75)	81 (44.02)
Non-randomised	210 (57.07)	14 (58.33)	95 (59.38)	101 (54.89)
Missing/Uncertain/N.A.	5 (1.36)	0 (0.00)	3 (1.88)	2 (1.09)
Blinding				
Quadruple-Blind	6 (1.63)	0 (0.00)	2 (1.25)	4 (2.17)
Triple-Blind	7 (1.90)	0 (0.00)	3 (1.88)	4 (2.17)
Double-blind	4 (1.09)	1 (4.17)	2 (1.25)	1 (0.54)
Single blind	14 (4.08)	2 (8.33)	7 (4.38)	5 (2.72)
Open label	336 (91.30)	21 (87.50)	145 (90.63)	170 (92.40)
Missing/Uncertain/N.A.	1 (0.27)	0 (0.00)	1 (0.63)	0 (0.00)
Sex/Age				
Women only	52 (14.13)	2 (8.33)	22 (13.75)	28 (15.22)
Men only	21 (5.71)	2 (8.33)	11 (6.88)	8 (4.35)
Both	295 (80.16)	20 (83.33)	127 (79.38)	148 (80.43)
Missing	0 (0.00)	0 (0.00)	0 (0.00)	0 (0.00)
Included children (<18 years)	51 (13.86)	3 (12.5)	19 (10.33)	19 (10.33)
Excluded elderly (>65 years)	25 (6.79)	1 (4.17)	10 (5.44)	25 (6.79)
Lead sponsor				
Industry	137 (37.23)	8 (33.33)	66 (41.25)	63 (34.24)
NIH	4 (1.09)	1 (4.17)	1 (0.63)	2 (1.09)
US Federal	0 (0.00)	0 (0.00)	0 (0.00)	0 (0.00)
Hospital and similar institutions	113 (30.71)	8 (33.33)	53 (33.13)	52 (28.26)
Universities and similar institutions	127 (34.51)	7 (29.17)	56 (35.00)	64 (34.78)
Other	19 (5.16)	1 (4.17)	3 (1.88)	15 (8.15)
Region				
Africa	6 (1.63)	0 (0.00)	3 (1.86)	3 (1.63)
Asia and Pacific	194 (52.72)	81 (50.63)	81 (50.63)	100 (54.35)
Central and South America	12 (3.26)	4 (2.50)	4 (2.50)	7 (3.80)
Europe	63 (17.12)	24 (15.00)	24 (15.00)	36 (19.57)
Middle East	5 (1.36)	2 (1.25)	2 (1.25)	3 (1.63)
North America	66 (17.93)	31 (19.38)	31 (19.38)	31 (16.85)
Missing	38 (10.32)	16 (10.0)	16 (10.00)	19 (10.33)
Study registration				
Start before submission	145 (39.40)	8 (33.33)	64 (40.00)	73 (39.67)
Start after submission	223 (60.60)	16 (66.67)	96 (60.00)	111 (60.33)
Data Monitoring Committee				
Yes	182 (49.46)	4 (16.67)	44 (27.5)	58 (31.52)
No	107 (29.08)	13 (54.17)	85 (53.13)	85 (46.20)
Missing/Unclear	79 (21.47)	7 (29.17)	31 (19.38)	41 (22.28)

* The sections for interventions, lead sponsor, and region do not add up to 100% because, in some included studies, numerous interventions were analyzed at once, multiple potential sponsors are single in a single phase IV clinical trial, or the investigations were performed in multiple locations.

**Table 3:** Characteristics of the included phase IV clinical trials by type of anti-cancer pharmacologic agent *.

	Number (%)			
	Chemotherapy (*n* = 158)	Immunotherapy (*n* = 22)	Hormonal Therapy (*n* = 65)	Targeted Therapy (*n* = 197)
Overall status *				
Not yet recruiting	11 (6.96)	2 (9.09)	3 (4.62)	15 (7.61)
Recruiting	37 (23.42)	4 (18.18)	19 (29.23)	53 (26.90)
Completed	25 (15.82)	3 (13.64)	17 (26.15)	42 (21.32)
Suspended	0 (0.00)	0 (0.00)	0 (0.00)	1 (0.51)
Terminated	10 (6.33)	2 (9.09)	5 (7.69)	12 (6.09)
Withdrawn	1 (0.63)	1 (4.55)	4 (6.15)	4 (2.03)
Active. not recruiting	6 (3.80)	3 (13.64)	7 (10.77)	24 (12.18)
Enrolling by invitation	0 (0.00)	0 (0.00)	0 (0.00)	3 (1.52)
Enrollment				
1–299	130(82.28)	19(86.36)	54(83.08)	171(86.80)
300–599	19(12.03)	1(4.55)	6(9.23)	11(5.58)
600–2999	8(5.06)	1(4.55)	1(1.54)	11(5.58)
≥3000	0(0.00)	0(0.00)	0(0.00)	0(0.00)
Intervention model				
Crossover assignment	2 (1.27)	2 (9.09)	1 (1.54)	0 (0.00)
Single group assignment	58 (36.71)	9 (40.91)	33 (50.77)	125 (63.45)
Parallel assignment	94 (59.49)	10 (45.45)	30 (46.15)	69 (35.03)
Factorial assignment	0 (0.00)	0 (0.00)	0 (0.00)	0 (0.00)
Missing	4 (2.53)	1 (4.55)	1 (1.54)	3 (1.52)
Allocation				
Randomised	86(54.43)	8(36.36)	27 (41.54)	57 (28.93)
Non-randomised	69 (43.67)	13(59.09)	38(58.46)	138(70.50)
Missing/Uncertain/N.A.	3(1.90)	1 (4.55)	0(0.00	2 (1.02)
Blinding				
Quadruple-Blind	2 (1.27)	0 (0.00)	1 (1.54)	2 (1.02)
Triple-Blind	3 (1.90)	0 (0.00)	3 (4.62)	2 (1.02)
Double-blind	1 (0.63)	0 (0.00)	0 (0.00)	2 (1.02)
Single blind	6 (3.80)	1 (4.55)	4 (6.15)	5 (2.54)
Open label	146 (92.50)	21(95.45)	56 (86.15)	186 (94.42)
Missing/Uncertain/N.A.	0(0.00)	0 (0.00)	1 (1.54)	0(0.00)
Sex/Age				
Women only	28(17.72)	0(0.00)	17(26.15)	19(9.64)
Men only	4(2.53)	0(0.00)	15(23.08)	2(1.02)
Both	126(79.75)	22(100.00)	33(50.77)	176(89.34)
Missing	0(0.00)	0(0.00)	0(0.00)	0(0.00)
Included children (<18 years)	26(16.46)	2(9.09)	8(12.31)	23(11.68)
Excluded elderly (>65 years)	12(7.59)	0(0.00)	3(4.62)	6(3.05)
Lead sponsor				
Industry	38 (24.05)	6 (27.27)	30 (46.15)	95 (48.22)
NIH	1 (0.63)	0 (0.00)	3 (4.62)	0 (0.00)
US Federal	0 (0.00)	0 (0.00)	0 (0.00)	0 (0.00)
Hospital and similar institutions	51 (32.28)	7 (31.82)	14 (21.54)	58 (29.44)
Universities and similar institutions	72 (45.57)	11 (50.00)	19 (29.23)	52 (26.4)
Other	8 (5.06)	0 (0.00)	3 (4.62)	11 (5.58)
Region				
Africa	3 (1.90)	1 (4.55)	0 (0.00)	3 (1.52)
Asia and Pacific	84 (53.16)	11 (50.00)	36 (55.38)	91 (46.19)
Central and South America	4 (2.53)	0 (0.00)	3 (4.62)	9 (4.57)
Europe	29 (18.35)	6 (27.27)	11 (16.92)	39 (19.8)
Middle East	3 (1.90)	0 (0.00)	1 (1.54)	3 (1.52)
North America	29 (18.35)	2 (9.09)	10 (15.38)	37 (18.78)
Missing	15 (9.49)	2 (9.09)	5 (7.69)	24 (12.18)
Study registration				
Start before submission	78 (49.37)	12 (54.55)	21 (32.31)	71 (36.04)
Start after submission	80 (50.63%	10 (45.45)	44 (67.69)	126 (63.96)
Data Monitoring Committee				
Yes	51 (32.28)	8 (36.36)	21 (32.31)	49 (24.87)
No	71 (44.94)	9 (40.91)	44 (67.69)	107 (54.32)
Missing/Unclear	36 (22.78)	5 (22.73)	0 (0.00)	41 (20.81)

* Data concerning the herbal/traditional (*n* = 14) and device (*n* = 2) studies were not included here due to the limited number of studies.

**Table 4:** The distribution of pharmacologic intervention by type of cancer.

	Number (%)			
	Chemotherapy (*n* = 158)	Immunotherapy (*n* = 22)	Hormonal Therapy (*n* = 65)	Targeted Therapy (*n* = 197)
Cancer Type				
Blood	1 (0.63)	15 (68.18)	17 (26.15)	61 (30.96)
Solid Tumors	114 (43.51)	7 (2.67)	46 (17.56)	129 (49.24)
Lung	26 (16.46)	1 (4.55)	1 (1.54)	50 (25.38)
Hepatobiliary/pancreatic	10 (6.33)	0 (0.00)	2 (3.08)	15 (7.61)
Gastrointestinal	10 (6.33)	1 (4.55)	3 (4.62)	14 (7.11)
Breast	52 (32.91)	0 (0.00)	23 (35.38)	20 (10.15)
Endocrine	4 (2.53)	0 (0.00)	2 (3.08)	2 (1.02)
Genitourinary	4 (2.53)	0 (0.00)	0 (0.00)	6 (3.05)
Reproductive	24 (15.19)	0 (0.00)	17 (26.15)	5 (2.54)
Head and Neck	3 (1.90)	1 (4.55)	0 (0.00)	3 (1.52)
Central Nervous System	1 (0.63)	0 (0.00)	1 (1.54)	6 (3.05)
Skin	0 (0.00)	2 (9.90)	0 (0.00)	6 (3.05)
Other	5 (3.17)	1 (4.55)	3 (4.62)	12 (6.09)

## Data Availability

The data used in this study are freely available from ClinicalTrials.gov.

## Supplementary Materials

The following supporting information can be downloaded at: https://www.mdpi.com/article/10.3390/curroncol30060443/s1. List of included studies with their NCT number and title.
