# Supplementary material for: Characteristics of Phase IV Clinical Trials in Oncology: An Analysis Using the ClinicalTrials.gov Registry Data
Source: Curr Oncol. 2023 Jun 20;30(6):5932–45. doi: 10.3390/curroncol30060443 (PMC10297460; doi:10.3390/curroncol30060443)
Supplement: Supplementary file 1 [file curroncol-30-00443-s001.zip › curroncol-2408218-supplementary.pdf]

NCT NUMBER	TITLE
NCT01961544	Eribulin Mesylate Phase IV Clinical Trial in Korean Patients With Metastatic or Locally Advanced Breast Cancer
NCT03111615	HOrmone Therapy Immediately After Histological Diagnosis of Breast Cancer
NCT03583944	A Study to Evaluate Safety, Tolerability and Efficacy of Eribulin Mesylate in Treating Adult Females With Locally Advanced or Metastatic Breast Cancer
NCT03573960	A Study to Evaluate the Safety and Efficacy of Lenvatinib in Participants With Refractory Differentiated Thyroid Cancer
NCT03220178	Impact of eHealth-support on Quality of Life in Metastatic Breast Cancer Patients Treated With Palbociclib and Endocrine Therapy
NCT02549677	Epirubicin Versus Docetaxel Plus Cyclophosphamide in Lymph Node Negative, ER-positive, Her2-negative Breast Cancer
NCT04108208	A Study of Apalutamide in Chinese Participants With Non Metastatic Castration Resistant Prostate Cancer (NM-CRPC)
NCT01849380	Neoadjuvant ECS Versus ECF in Local Advanced Breast Cancer
NCT02679755	Palbociclib In Combination With Letrozole As Treatment Of Post-Menopausal Women With HR+, HER2- Advanced Breast Cancer
NCT04707196	A Study of Abemaciclib in Indian Women With Advanced Breast Cancer
NCT04929548	Exploratory Study of Neoadjuvant Treatment of HER2-positive Breast Cancer With Py in Combination With HP
NCT03035032	A Study of ELIGARD® in Hormone-dependent Prostate Cancer Patients
NCT05348876	A Study to Learn More About How Safe Darolutamide is and How Well it Works Under Real World Conditions When Taken in Addition to Standard Androgen Deprivation Therapy (ADT) in Indian Participants With High-risk Non-metastatic Castration-resistant Prostate Cancer (nmCRPC)
NCT04507035	Treating Locally Advanced Head and Neck Malignant Tumor With Anlotinib and Chemoradiotherapy
NCT04747951	Total Neoadjuvant Therapy in Rectal Cancer Treatment
NCT03123770	Efficacy and Safety of Neoadjuvant DC-T in Breast Cancer Patients
NCT01739764	An Extension (Rollover) Study of Vemurafenib in Participants With BRAF V600 Mutation-Positive Malignancies Previously Enrolled in an Antecedent Vemurafenib Protocol
NCT02918968	Study on Enzalutamide and Flutamide in Patients With Castration Resistant Prostate Cancer
NCT04164797	Study of Endostar Combined With TP Regimen for Esophageal Cancer

NCT02557503	Hepatic Arterial Infusion of Oxaliplatin and Fluorouracil Treatment of Advanced Primary Liver Cancer After TACE
NCT04330040	Prospective Multicentre Phase-IV Clinical Trial of Olaparib in Indian Patients With Ovarian and Metastatic Breast Cancer
NCT05420454	A Study for the Neoadjuvant Treatment of Breast Cancer
NCT05420467	A Study for the Adjuvant Treatment of Breast Cancer
NCT02089854	Adjuvant Endocrine Therapy for Estrogen Receptor-beta Positive Triple Negative Breast Cancer
NCT02199327	Topical Interferon Alfa 2b and Mitomycin C in Conjunctival-Corneal Intraepithelial Neoplasia
NCT01959061	Efficacy and Safety of Raltitrexed-based Transarterial Chemoembolisation(TACE)for Colorectal Cancer Liver Metastases
NCT05457257	Clinical Study to Assess the Efficacy and Safety of Olaparib in Chinese Patients With Metastatic Castration-Resistant Prostate Cancer Who Have Failed Prior Treatment With a New Hormonal Agent and Have BRCA1/2 Mutations
NCT05033769	Assessing ImmunoResponse Post Eribulin: Eribulin and Immunogenicity in Advanced Breast Cancer
NCT05148767	UGT1A1-Based Irinotecan Therapy for Locally Advanced Rectal Cancer
NCT05183126	Pharmacokinetic Study of Skeletal Muscle Area-based Paclitaxel Infusion in Patients With Breast Cancer
NCT02502864	Multicenter Study of Pharmacokinetic-Guided Docetaxel in Breast Cancer Patients Receiving Docetaxel and Cyclophosphamide
NCT04136782	Albumin-bound Paclitaxel and Carboplatin Versus Epirubicin and Docetaxel for Triple-negative Breast Cancer
NCT05291910	Inetetamab Combined With Anti-PD-1 Monoclonal Antibody and Albumin-Bound Paclitaxel for HER2+ Metastatic Breast Cancer
NCT04921137	Evaluating Harms and Benefits of Endocrine Therapy in Patients ≥70 Years of Age With Lower Risk Breast Cancer
NCT05507398	The Anti-tumor Effect of Metformin And Atorvastatin in Breast Cancer
NCT05191914	Clinical Study of Fulvestrant Combined With Chidamide in the Treatment of Hormone Receptor-positive Advanced Breast Cancer Resistant to CDK4/6 Inhibitors
NCT04182568	Nab-paclitaxel Compared With Docetaxel Followed by Anthracyclines and Cyclophosphamide in the Neoadjuvant Breast Cancer
NCT05362760	Combination of Abemaciclib and Endocrine Therapy in Hormone Receptor Positive HER2 Negative Locally Advanced or Metastatic Breast Cancer With Focus on Digital Side Effect Management
NCT04928261	Evaluating 6-months of HER2-targeted Therapy in Patients With HER2 Positive Early-stage Breast Cancer That Achieve a

	Pathological Complete Response to Neoadjuvant Systemic Therapy
NCT02945267	Nimotuzumab Plus S1 Versus Placebo Plus S1 as Maintenance Treatment in Patients With Unresectable Pancreatic Cancer
NCT03591549	Fulvestrant in Metastatic Breast Cancer
NCT02484677	Pharmacokinetics and Pharmacogenetics-based Adaptive Dosing of 5-fu (5-Fluorouracile) in Head & Neck Cancer Patient Undergoing Docetaxel, Cisplatin, 5-Fluorouracile (=TPF) Therapy
NCT05525481	Tamoxifen Prediction Study in Patients With ER+ Breast Cancer
NCT02301962	Phase IV Panitumumab Study in Indian Subjects With Metastatic Colorectal Cancer
NCT05580354	Safety and Efficacy of BCG Combined With Tislelizumab for BCG-untreated Patients With High-risk Non-muscle Invasive Bladder Cancer
NCT02344940	Safety of Toremifene and Tamoxifen Therapy in Premenopausal Patients With Operable Breast Cancer
NCT02903524	Doxorubicin Hydrochloride Liposome Injection Combination With Cyclophosphamide vs Pirarubicin Combination With Cyclophosphamide in Patients With Locally Advanced Breast Cancer
NCT02419742	Safety and Efficacy of Trastuzumab as Part of Breast Cancer Treatment Regimen
NCT03843229	Clinical Trial of Cinobufacini Combined With Transarterial Chemoembolization (TACE) on Primary Liver Cancer
NCT02445586	Safety Study of Pertuzumab (in Combination With Trastuzumab and Docetaxel) in Indian Participants With Breast Cancer
NCT02776527	A Clinical Trial of Maintenance Treatment of Apatinib in Advanced Gastric Cancer Patients Have Completed Postoprative Adjuvant Chemotherapy
NCT04813913	VASCular Impact of Angiogenic Treatment in Patients With Advanced Colorectal Cancer
NCT05631795	Study to Assess the Safety of Alpelisib Plus Fulvestrant, in Men and Post-menopausal Women With HR-positive, HER2-negative, Advanced Breast Cancer (aBC) With PIK3CA Mutation, Whose Disease Progressed on or After Endocrine Treatment
NCT03988114	A Study of Abemaciclib (LY2835219) in Participants With HR+, HER2- Advanced Breast Cancer
NCT03641560	A Safety and Efficacy Study of Enzalutamide in Indian Patients With Progressive Metastatic Castration-Resistant Prostate Cancer (mCRPC) Previously Treated With Docetaxel-Based Chemotherapy
NCT03799679	Albumin-Bound Paclitaxel Followed by Epirubicin in Combination With Cyclophosphamide in Triple Negative Breast Cancer

NCT04031885	A Study of Abemaciclib (LY2835219) in Combination With Fulvestrant Compared to Chemotherapy in Women With HR Positive, HER2 Negative Metastatic Breast Cancer
NCT03948568	Evaluating Optimal Timing of Endocrine Therapy and Radiation Therapy in Early-stage Breast Cancer (REaCT-RETT)
NCT02405858	A Study of Abiraterone Acetate in Metastatic Castration-Resistant Prostate Cancer Participants Who Responded Poorly to the First-line Combined Androgen Blockade Therapy
NCT01743560	An Open Label Study of Postmenopausal Women With Oestrogen Receptor Positive Locally Advanced or Metastatic Breast Cancer Treated With Everolimus (RAD001) With Exemestane, With Exploratory Epigenetic Marker Analysis
NCT03270007	Research of Intensive Treatment in Hormone Receptor<10% and Human Epidermal Growth Factor Receptor-2 Negative Breast Cancer Patients With Positive Lymph Node Residual Disease After Neoadjuvant Chemotherapy
NCT03664687	Comparing a Single-Dose vs. Twice Yearly Zoledronate in Patients With Early Stage Breast Cancer (REaCT-ZOL)
NCT02726009	A Trial to Evaluate Safety of Firmagon® (Degarelix) in Indian Patients Diagnosed With Advanced Hormone-dependent Prostate Cancer
NCT02207361	Paclitaxel in Combination With Carboplatin Versus Paclitaxel Plus Epirubicin in Metastatic Breast Cancer
NCT03355157	A Randomized, Open-label, Multi-center Phase IV Study Evaluating Palbociclib Plus Endocrine Treatment Versus a Chemotherapy-based Treatment Strategy in Patients With Hormone Receptor Positive / HER2 Negative Breast Cancer in a Real World Setting (GBG 93 - PADMA Study).
NCT03799692	Albumin-Bound Paclitaxel Combined With Carboplatin as Neoadjuvant Chemotherapy in Luminal B/HER-2 Negative Breast Cancer
NCT03971110	A Study of Neoadjuvant Hormone Therapy in Patient With Advanced Prostate Cancer Undergoing Radical Prostatectomy.
NCT02869789	An Investigational Immuno-therapy Study for Safety of Nivolumab in Combination With Ipilimumab to Treat Advanced Cancers
NCT03274284	Chemoradiotherapy for Recurrent T1G3 Bladder Cancer
NCT02116582	A Study to Evaluate Enzalutamide After Abiraterone in Metastatic Castration-Resistant Prostate Cancer
NCT03790384	Sequential Combination Therapy in Bladder Cancer
NCT05035147	Albumin-bound Paclitaxel Combined With Gemcitabine First-line Inoperable Pancreatic Cancer
NCT02441517	A Study of Enzalutamide Re-treatment in Metastatic Castration-resistant Prostate Cancer After Docetaxel and/or Cabazitaxel Treatment
NCT02401971	Irinotecan Plus Thalidomide in Second Line Advanced Gastric Cancer

NCT01896479	A Study of Two Different Doses of Cabozantinib (XL184) in Progressive, Metastatic Medullary Thyroid Cancer
NCT02074137	Evaluation of Safety of Cabazitaxel (Jevtana) in Patients With Metastatic Hormone Refractory Prostate Cancer
NCT03645187	Celecoxib as Adjuvant Therapy to Chemotherapy in Patients With Metastatic Colorectal Cancer
NCT03691441	Comparative Effectiveness Trial of Transoral Head and Neck Surgery Followed by Adjuvant Radio(Chemo)Therapy Versus Primary Radiochemotherapy for Oropharyngeal Cancer
NCT04874207	Evaluation of Treatment PERSOnalization Based on Its Therapeutic Monitoring in Patients With Metastatic Colorectal Cancer Treated With REgorafenib
NCT05036005	Neoadjuvant Ontruzant (SB3) in Patients With HER2-positive Early Breast Cancer: An Open-Label (NeoON)
NCT02557490	Oxaliplatin and Raltitrexed Treatment of Colorectal Cancer With Liver Metastases
NCT03564938	Regorafenib in Indian Patients With Metastatic Colorectal Cancer (mCRC).
NCT04217096	Efficacy and Safety of Paclitaxel Liposome and S-1 as First-line Therapy in \ Advanced Pancreatic Cancer Patients
NCT05302336	AC vs TC in Patients With HR-positive, HER2-negative Early Breast Cancer
NCT04513522	A Study to Evaluate the Safety and Efficacy of Nivolumab With Ipilimumab in Participants With Untreated Advanced Kidney Cancer Conducted in India
NCT04810585	Plasma Biomarker for Aflibercept in Advanced Colorectal Cancer
NCT02195453	Chemotherapy Combined With Yangzhengxiaoji Capsule in Patients With Advanced Non-Small Cell Lung Cancer
NCT04526704	Study to Evaluate Discontinuation and Re-Treatment in Participants With Tenosynovial Giant Cell Tumor (TGCT) Previously Treated With Pexidartinib
NCT04511533	Dacomitinib for Treatment of Patients in India With Metastatic Non Small Cell Lung Cancer With EGFR Activating Mutations
NCT01914692	Application of Somatostatin for Advanced Gastric Cancer After D2 Lymph Node Dissection
NCT04319198	Rollover Study in Participants With Metastatic Solid Tumors Benefiting From Therapy With Sacituzumab Govitecan-hziy
NCT01994031	Dose Escalation and Pharmacokinetic Study of Paclitaxel Liposome Injection in Treating Patients With Advanced Solid Tumor After Failure From Conventional Treatments
NCT03289741	A Study to Evaluate Patient Experience in the Therapy of Neuroendocrine Tumors Treated With Octreotide Long Acting Release Versus Lanreotide
NCT02691793	Study to Evaluate the Safety and Efficacy of Sunitinib, in Subject With Refractory Solid Tumors

NCT02688881	Study to Evaluate the Safety and Efficacy of Sirolimus, in Subject With Refractory Solid Tumors
NCT05131841	Cipterbin Combined With Vinorelbine in the Treatment of HER2-positive MBC
NCT03927391	Effect of a Reduced Dose Enzalutamide in Frail (m)CRPC Patients on Cognitive Side Effects
NCT02779257	Pasireotide Treatment for Neuroendocrine Tumor
NCT03980704	Preoperative High Protein vs Immunodiet in Surgical Cancer Patients
NCT03890055	Clinical Study of First-line Treatment of Small Cell Lung Cancer (SCLC) With Anlotinib Hydrochloride
NCT01962376	Preoperative Chemotherapy With Bevacizumab For Potentially Resectable Gastric Cancer With Liver Metastasis
NCT02380131	Perioperative Chemotherapy With Herceptin For Potentially Resectable HER-2 Positive Gastric Cancer With Liver Metastasis
NCT05312840	Efficacy and Safety of Conventional and Low-dose Platinum Gemcitabine Combined With Cindilimab With Delayed Administration in First-line Treatment of Advanced Squamous Non-small Cell Lung Cancer
NCT05187208	PARP Inhibitor Oral Maintenance in Low-Risk Ovarian Cancer
NCT02348450	Irinotecan Plus Cisplatin Compared With Etoposide Plus Cisplatin for Extensive Stage Small-cell Lung Cancer
NCT03264794	the Clinical Trial of Gefitinib(Non - Small Cell Lung Cancer)
NCT05250648	Clinical Trial on HIPEC With Mitomycin C in Colon Cancer Peritoneal Metastases (GECOP-MMC)
NCT01932125	An Interventional Study of Avastin (Bevacizumab) in Patients With Advanced/Metastatic Epithelial Ovarian Cancer, Fallopian Tube Cancer or Primary Peritoneal Cancer
NCT04541706	Lorlatinib in ALK Inhibitor Treated Unresectable Advanced/Recurrent ALK-Positive Non Small Cell Lung Cancer Patients in India
NCT04814056	To Evaluate the Efficacy of Afatinib in the Treatment of Locally Advanced/Metastatic Non-Small Cell Lung Cancer With NRG1 Fusion
NCT05071703	Evaluation of TRILACICLIB in Chinese Patients With Extensive-stage Small Cell Lung Cancer (ES-SCLC) for Chemotherapy-induced Myelosuppression, Antitumor Effects of Combination Regimens, and Safety in a Real-world Study
NCT01977651	A Study to Evaluate the Potential Increased Risk of Seizures Among Metastatic Castration-Resistant Prostate Cancer (mCRPC) Patients Treated With Enzalutamide
NCT02996214	Paclitaxel Liposome for Squamous Non-Small-cell Lung Cancer Study(LIPUSU)
NCT01720901	Increased Dose of Icotinib in Advanced None Small Cell Lung Cancer Patients After Routine Gefitinib Therapy

NCT02031601	Intercalated Combination of Chemotherapy and Tyrosine Kinase Inhibitors as First-line Treatment for Patients With Non-Small-Cell Lung Cancer
NCT01789281	Everolimus Roll-over Protocol for Patients Who Have Completed a Previous Novartis-sponsored Everolimus Study.
NCT03792503	Pemetrexed Plus Apatinib Maintenance Treatment in Patients With Non-squamous Non-small Cell Lung Cancer Patients Who Have Not Progressed After 4 Cycles of Induction Chemotherapy of Pemetrexed in Combination With Platinum-based Regimen
NCT02627248	Neoadjuvant Chemotherapy With or Without Huaier Granule in Treating Women With Locally Advanced Breast Cancer That Can Be Removed By Surgery
NCT02142790	Weekly and Every 3 Week Administration of Paclitaxel Liposome Injection in Metastatic Breast Cancer
NCT04137640	Palbociclib + Letrozole Versus Epirubicin + Cyclophosphamide and Sequential Docetaxel as Neoadjuvant Chemotherapy
NCT02744664	Cryotherapy Combine Icotinib for Advanced NSCLC Treatment
NCT02615457	Huaier Granule in Treating Women With Triple Negative Breast Cancer
NCT02513355	Endostar Treatment of Advanced Non-small Cell Lung Cancer Multi-center Clinical Research
NCT02584933	Roll-over Study to Allow Access to Certinib (LDK378) for Patients Who Are on Ceritinib Treatment in a Novartis-sponsored Study
NCT01972490	Avastin in Combination With Chemotherapy for RAS Mutant Unresectable Colorectal Liver-limited Metastases
NCT05452213	Comprehensive Analysis of Spatial, Temporal and Molecular Patterns of Ribociclib Efficacy and Resistance in Advanced Breast Cancer Patients
NCT03844360	Dose Individualization of Antineoplastic Drugs and Anti-Infective Drug in Children With Hematoplastic Disease
NCT05304169	Study to Evaluate the Safety and Efficacy of Switching From Zoladex® Monthly or Quarterly, to Eligard® Semiannual.
NCT02575378	Maintenance Treatment With Capecitabine Metronomic Chemotherapy and Chinese Traditional Medicine in Metastatic Colorectal Cancer
NCT03752216	NIraparib and Quality Of LifE is a Longitudinal Study Evaluating in Real Life the Tolerability of Niraparib.
NCT02485691	Cabazitaxel Versus the Switch to Alternative AR-targeted Agent (Enzalutamide or Abiraterone) in Metastatic Castration-resistant Prostate Cancer (mCRPC) Patients Previously Treated With Docetaxel and Who Rapidly Failed a Prior AR-targeted Agent
NCT03853551	Osimertinib Study in Indian Patients
NCT03432949	Radium-223 Combined With Dexamethasone as First-line Therapy in Patients With M+CRPC

NCT02860429	Clinical Trial of Cinobufacini Injection Combined With Oxaliplatin Regimen on Gastrointestine Carcinoma
NCT02875314	HeadStart4: Newly Diagnosed Children (<10 y/o) With Medulloblastoma and Other CNS Embryonal Tumors
NCT03130634	The Efficacy of Silymarin as Adjuvant Therapy on Colorectal Cancer Patients Undergoing FOLFIRI Treatment
NCT04331626	Low-dose Gemcitabine Combined With Nivolumab for Second-line and Above Line Treatment of NSCLC
NCT03127072	Study Comparing Radio Frequency Ablation Plus Chemotherapy and Chemotherapy Alone in Patients With Unresectable CRLM
NCT02316327	Open-label Study in Patients With Metastatic NSLC Treated With Cisplatin, Gemcitabine and Bevacizumab
NCT02480634	Zoledronic Acid Combined Radiotherapy for Bone Metastasis of Non-small Cell Lung Cancer
NCT03164980	Comparison of QoL Between Trabectedin/PLD and Standard Platinum-based Therapy in Patients With Platinum Sensitive Recurrent Ovarian Cancer
NCT04864405	Evaluating the Dose Timing (Morning vs Evening) of Endocrine Therapy and Its Effects on Tolerability and Compliance
NCT03238703	Endocrine Therapy in Treating Patients With HER2 Negative, Low Risk Breast Cancer
NCT03384511	The Use of 18F-ALF-NOTA-PRGD2 PET/CT Scan to Predict the Efficacy and Adverse Events of Apatinib in Malignancies.
NCT03401827	The Effect of Gemcitabine Plus Nab-paclitaxel as Secondary Chemotherapy in Advanced Pancreatic Cancer
NCT05098990	Jinfukang Oral Liquid Combined With Chemotherapy for Treating Driver Gene-negative Advanced NSCLC
NCT04601441	Study to Evaluate ctDNA of mCSPC Patients Receiving Apalutamide in Japan
NCT04416633	Study to Access Safety of Durvalumab in Indian Adult Patients With Locally Advanced, NSCLC
NCT05525767	Chemotherapy Combined With Bevacizumab Neoadjuvant Therapy for HER2-negative Breast Cancer
NCT03444766	Study of Nivolumab for Advanced Cancers in India
NCT05161195	Roll-over Study to Allow Continued Access to Ribociclib
NCT02151149	Safety and Efficacy Study of Abraxane in Combination With Carboplatin to Treat Advanced NSCL Cancer in the Elderly
NCT02842749	Phase IV Study of the Safety and Efficacy of Everolimus in Adult Patients With Progressive pNET in China
NCT04767828	A Single Arm Study of Brain Metastasis in Patients With HER2-positive Breast Cancer
NCT05144997	Lorlatinib Continuation Study
NCT03794778	Evaluation of PLD Combined With Carboplatin Versus Paclitaxel Plus Carboplatin in the First-line Treatment of Epithelial Ovarian Cancer

NCT03986528	Clinical Trial of Kanglaite Injection (KLTi) in Advanced Non-Small Cell Lung Cancer (NSCLC)
NCT03413436	Lobaplation or Cisplatin in Adjuvant Chemotherapy for Esophageal Carcinoma
NCT03348969	Neoadjuvant Short-term Intensive Chemoresection Versus Standard Adjuvant Intravesical Instillations in NMIBC
NCT02194556	Sequential and Maintenance Icotinib Plus Chemotherapy Versus Icotinib Maintenance After Chemotherapy in Advanced NSCLC
NCT05229809	Clinical Trial of Yiqi Wenyang Jiedu Prescription in the Prevention and Treatment of Postoperative Metastasis and Recurrence of Gastric Cancer
NCT04356118	Efficacy and Safety of Recombinant Human Endostatin in Non-Small Cell Lung Cancer With Leptomeningeal Metastasis
NCT02283424	Icotinib as the Adjunctive Treatment After Surgery in Stage I-IIIB Lung Adenocarcinoma Patients With EGFR Gene Mutation
NCT03998696	An Experimental Study to Compare Treatment Response and Toxicities of Concurrent Chemoradiation With Weekly Cisplatin and Three Weekly Cisplatin in Locally Advanced Head and Neck Cancer.
NCT04356222	Efficacy and Safety of Durvalumab in Non-Small Cell Lung Cancer With Leptomeningeal Metastasis
NCT01926171	Icotinib With Whole Brain Radiation Therapy in NSCLC Patients With Brain Metastases
NCT02015650	Cetuximab Compared to Mitomycin-C and 5-Fluorouracil for Locally Advanced Squamous Cell Carcinomas of the Head and Neck
NCT02739360	Roll Over Study to Provide Idelalisib to Participants Previously Treated With the Investigational PI3Kδ Inhibitor, GS-9820
NCT03083210	Study of Lanreotide in Metastatic or Recurrent Grade I-II Hindgut NET
NCT04401059	Synergistic Effect of Elemene Plus TKIs Compared With TKIs in EGFR-mutated Advanced NSCLC：Prospective Study
NCT01849588	HCV-RNA Kinetics During Sorafenib for Hepatocellular Carcinoma (HCC)
NCT04790305	Effect of Huaier Granule on Adjuvant Treatment for High-risk Early-stage Triple-negative Breast Cancer
NCT03655821	Dose Individualization of Pemetrexed - IMPROVE-II
NCT04825574	Study for Patients Previously Treated in Avapritinib Clinical Trials
NCT04127396	Lenvatinib Plus TACE Versus Sorafenib Plus TACE for HCC With PVTT
NCT05183828	Effect of HSD3B1 (1245C) Gene Mutation on Treatment of Stage I-III Breast Cancer
NCT03673332	Elderly Cancer PatIents, Safety and qualiTy of Life Under immunOtheraPies
NCT05320406	RElugolix VErsus LeUprolide Cardiac Trial

NCT04269369	Implementation of Pre-emptive Geno- and Phenotyping in 5-Fluorouracil- or Capecitabine-treated Patients
NCT05488353	A Clinical Study of Disitamab Vedotin for Injection Combined With Penpulimab Injection in Neoadjuvant Therapy for Patients With HER2-expressing Cisplatin-intolerant cT2-T4aNxM0 Bladder Urothelial Carcinoma
NCT05106179	The Efficacy and Safety of Beta-blockers Drugs in Adults With Spinal Hemangioma
NCT03891979	Gut Microbiome Modulation to Enable Efficacy of Checkpoint-based Immunotherapy in Pancreatic Adenocarcinoma
NCT03908138	RDD Versus VDD in Newly Diagnosed Patients With Multiple Myeloma
NCT02426034	A Study of Apatinib Tablets in the Treatment of Advanced or Metastatic Gastric Cancer
NCT03607149	Pemetrexed in Maintenance in Patients With Impaired Renal Function, 2 Dose Calculation Strategies
NCT05490953	Enhancing Effect on Tumour Apoptosis With the Use of Pentoxifylline in Patients With Hodgkin Lymphoma
NCT05682131	South China Children Cancer Group - Relapsed-Acute Lymphoblastic Leukemia 2022 Protocol
NCT05387109	Penpulimab Combined With Anlotinib in Neoadjuvant Treatment of Resectable Non-small Cell Lung Cancer
NCT04362072	Study of Lorlatinib In People With ALK-positive Non-small Cell Lung Cancer
NCT02404675	High Dose Icotinib in Advanced Non-small Cell Lung Cancer With EGFR 21 Exon Mutation
NCT03460678	Randomized Comparative Study of Erlotinib and Pemetrexed in the Maintenance Treatment of Advanced Lung Cancer Patients.
NCT03092986	Outcome of Cisplatin and Vinblastine Versus Paclitaxel and Carboplatin as Sequential Chemotherapy Followed by Radiotherapy in Locally Advanced Non-small Cell Lung Cancer
NCT03715205	Study to Evaluate the Safety of Pembrolizumab in Participants With Unresectable or Metastatic Melanoma or Non-small Cell Lung Cancer in India (MK-3475-593/KEYNOTE-593)
NCT03873311	Azacytidine + HAG Regimen vs. Azacytidine for Elderly Patients With Newly Diagnosed Myeloid Malignancy
NCT03285763	A Study of Atezolizumab (Tecentriq) to Investigate Long-term Safety and Efficacy in Previously-treated Participants With Locally Advanced or Metastatic Non-small Cell Lung Cancer (NSCLC)
NCT02208843	Afatinib as Second-line Therapy for Lung Cancer With Epidermal Growth Factor Receptor (EGFR) Mutation
NCT03340506	Dabrafenib and/or Trametinib Rollover Study
NCT04683575	Clinical Study on the Effect of Selenium Yeast Capsule on Prognosis of Differentiated Thyroid Carcinoma
NCT04078152	Durvalumab Long-Term Safety and Efficacy Study
NCT02467686	Breast Cancer, Sexuality and Black Cohosh

NCT05078671	Pharmacokinetic Boosting of Olaparib to Improve Exposure, Tolerance and Cost-effectiveness
NCT03768063	A Study in Patients Previously Enrolled in a Genentech and/or F. Hoffmann-La Roche Ltd Sponsored Atezolizumab Study
NCT05785208	Efficacy Study of Osimertinib in Treatment-naïve Patients With EGFR Mutant NSCLC According to TP53 Mutational Status.
NCT03148418	A Study in Participants Previously Enrolled in a Genentech- and/or F. Hoffmann-La Roche Ltd-Sponsored Atezolizumab Study (IMbrella A)
NCT02815319	Schedules of Dexamethasone in Patients Incorrectly Taking Dexamethasone Premedication (REaCT-dexamethasone)
NCT02103244	Validation of an Adjusted Dosing Algorithm of Carboplatin
NCT04641819	Yangzheng Compound Mixture in the Treatment of Sleep Disorder in Cancer Patients
NCT02812992	Geriatric Assessment Directed Trial to Evaluate Gemcitabine +/- Nab-paclitaxel in Elderly Pancreatic Cancer Patients
NCT05203172	The FLOTILLA Study: Providing Continued Access to The Study Medicines Encorafenib and Binimetinib for Participants in Prior Clinical Trials
NCT02000531	Progression Free Survival (PFS) Using Erlotinib for Non-Small-Cell Lung Cancer (NSCLC) in Chinese Population
NCT02299765	Intercalating and Maintenance Gefitinib in Combination With Chemotherapy for Advanced EGFR-mutant NSCLC
NCT05723991	Study of Disitamab Vedotin Combined With Gemcitabine in Neoadjuvant Treatment of Urothelial Carcinoma
NCT02103257	Sequential Icotinib Plus Chemotherapy Versus Icotinib Alone as First-line Treatment in Stage IIIB/IV Lung Adenocarcinoma
NCT05110196	Study of Capmatinib in Indian Patients With MET Exon 14 Skipping Mutation Positive Advanced NSCLC.
NCT03892330	Combination Therapy of Anthracyclines for Children With Nephroblastoma
NCT02081755	Safety and Efficacy of Everolimus Treatment in Liver Transplantation for Liver Cancer
NCT05722405	Ixazomib Plus Low-dose Lenalidomide Versus Ixazomib Alone for Maintenance Treatment of High Risk Multiple Myeloma
NCT05555329	Alternative Dosing Scheme of Pomalidomide 4 mg Every Other Day Versus Pomalidomide 2 mg and 4 mg Every Day; the POMAlternative Study
NCT05183139	A Multicenter In-class Transition Study of Ixazomib Combined With Pomalidomide and Dexamethasone or With Lenalidomide and Dexamethasone in Adults With Relapsed/Refractory Multiple Myeloma
NCT04989140	Study of Pomalidomide, Oral Dexamethasone and Ixazomib in Patients With Relapsed MM Who Have Received Lenalidomide
NCT02982954	A Study to Evaluate the Safety of Nivolumab and Ipilimumab in Subjects With Previously Untreated Advanced or Metastatic Renal Cell Cancer

NCT04348006	Assessment of Bortezomib (Alvocade ®) Efficacy and Safety in Newly Diagnosed Multiple Myeloma Patients
NCT04217967	Ixazomib, Lenalidomide, and Combination for Maintenance in NDMM Patients
NCT03934684	Study to Evaluate Safety Tolerability & Efficacy of Kyprolis (Carfilzomib) in Relapsed or Refractory Multiple Myeloma
NCT03829371	STUDY COMPARING TWO STANDARD TREATMENTS IN AUTOLOGOUS STEM CELL TRANSPLANTATION INELIGIBLE POPULATION AFFECTED BY MULTIPLE MYELOMA
NCT03768960	A Study of DARZALEX (Daratumumab) In Indian Participants With Relapsed and Refractory Multiple Myeloma, Whose Prior Therapy Included a Proteasome Inhibitor and an Immunomodulatory Agent
NCT03416374	A Study to Evaluate the Efficacy and Safety of Ixazomib in Combination With Lenalidomide and Dexamethasone in Patients With Relapsed and/or Refractory Multiple Myeloma Initially Treated With an Injection of Proteasome Inhibitor-Based Therapy
NCT03173092	A Study of Ixazomib (NINLARO®) in Combination With Lenalidomide and Dexamethasone (IRD) for the Treatment of Participants With Multiple Myeloma (MM)
NCT02773550	Treatment With a Scheme With Low Doses of Bortezomib / Melphalan / Prednisone (MPV) in Patients With Multiple Myeloma
NCT02577783	PDD vs PAD to Treat Initially Diagnosed MM
NCT02268890	A Pharmacokinetic Study of Bortezomib in Taiwanese Participants With Multiple Myeloma
NCT01868828	A Study of PAD Versus Velcade, Cyclophosphamide and Dexamethasone (VCD) Treatment in Subjects With Multiple Myeloma
NCT03712969	Shenlingcao Oral Liquid for Patients With Stage II or IIIA NSCLC
NCT03799601	Docetaxel Combined With Carboplatin Plus Anlotinib as First Line Treatment in NSCLC
NCT02778893	Conmana Combined With Thalidomide to Treat NSCLC
NCT03134456	Pembrolizumab for Metastatic NSCLC Patients Expressing PD-L1 Who Have Their Own PDX
NCT02514174	Afatinib Treatment for Patients With EGFR Mutation Positive NSCLC Who Are Age 70 or Older
NCT03672643	Long Term Safety Observation of Crizotinib in Chinese NSCLC Population
NCT02695290	Afatinib in EGFR+NSCLC (Recurrent or Stage IV) - Patients With Poor Performance Status (ECOG 2 or 3)
NCT04554524	Chemotherapy Combined With Pembrolizumab in Treating Patients With Thymoma and Thymic Carcinoma
NCT02929615	Study of Standard and Individualized Treatment Model for Relapse and Refractory Lymphatic System Malignant Tumors

NCT05634915	Clinical Study of rATG Individualized Administration in Haploidentical Hematopoietic Stem Cell Transplantation
NCT05144243	Study to Assess Adverse Events and Change in Disease State of Oral Venetoclax in Combination With Subcutaneous (SC) Azacitidine in Newly Diagnosed Adult Participants With Acute Myeloid Leukemia (AML) Who Are Ineligible for Intensive Chemotherapy in China
NCT05071482	Flumatinib Versus Imatinib Combined With Chemotherapy for de Novo Ph+ ALL
NCT03988205	Patients Receiving Induction With Liposomal Daunorubicin and Cytarabine (CPX-351) for Acute Myeloid Leukemia
NCT02926586	Fludarabine and Cytarabine Versus High-dose Cytarabine for CBF-AML
NCT05525338	Comparison of Standard Dose Alectinib to Alectinib in Adjusted Dose Based on Alectinib Bloodlevels
NCT05429515	Effect of HFR-SUPRA in the Treatment of Multiple Myeloma-related Acute Kidney Injury
NCT04506086	Feasibility Study to Evaluate Outpatient Blinatumomab in Subjects With Minimal Residual Disease (MRD) of B-precursor Acute Lymphoblastic Leukemia (ALL)
NCT03229200	Extended Treatment Protocol for Subjects Continuing to Benefit From Ibrutinib.
NCT05444673	A Prospective, Single-arm Clinical Study of Pabrolizumab in Combination With Cisplatin + 5-FU for the Neoadjuvant Treatment of Unresectable, Advanced Squamous Carcinoma of the Temporal Bone Single-arm Clinical Study
NCT04223583	Study on the Treatment of Soft Tissue Sarcoma With First-line Chemotherapy Failure by Anrotenil Hydrochloride Capsule
NCT03735758	Quality of Life in Patients With Non-adipocyte Soft Tissue Sarcoma Under Palliative Chemotherapy or Pazopanib
NCT03514381	Pharmacologic Interaction Between Ifosfamide and Aprepitant in Treated Patients With Soft Tissue Sarcoma
NCT01908972	The Safety and Efficiency of Propranolol as an Initial Treatment for Pediatric Hemangioma
NCT03975829	Pediatric Long-Term Follow-up and Rollover Study
NCT02068196	A National Phase IV Study With Ipilimumab for Patients With Advanced Malignant Melanoma.
NCT04056962	Tacrolimus for the Treatment of Superficial Kaposiform Hemangioendothelioma and Tufted Angioma
NCT05137860	Efficacy of the Use of Bortezomib for the Treatment of Relapsed Leukemia or Positive MRD
NCT04132102	To Evaluate the Efficacy of Afatinib in Advanced Lung Squamous Cell Carcinoma With EGFR Sensitive Mutation
NCT03725423	Apatinib for Advanced Lung Squmamous Carcinoma
NCT03610022	Relationship Between Pharmacokinetics and Safety of Vismodegib - OPTIVISMO-1

NCT02513342	Endostar First-line Treatment of Advanced Non-small Cell Lung Squamous Carcinoma
NCT02436408	VISmodegib for ORbital and Periocular Basal Cell Carcinoma
NCT02088515	Nedaplatin (Jiebaishu®) Combined With Docetaxel for Advanced Lung Squamous Cell Carcinoma
NCT01491711	Superficial Basal Cell Carcinoma Treatment With Topical Photodynamic Therapy With Fractionated 5-aminolevulinic Acid 20% Versus Two Stage Methylaminolevulinate
NCT05421897	Rapid Administration Pilot for Infusing Dinutuximab
NCT04406870	Sirolimus in the Treatment for Infantile Hepatic Hemangioendothelioma(IEEH)
NCT04480268	PAXG Out in the Country
NCT01990534	A Study of Brentuximab Vedotin in Participants With Relapsed or Refractory Hodgkin Lymphoma
NCT01856023	HD IL-2 + Ipilimumab in Patients With Metastatic Melanoma
NCT02631499	Efficacy Study of TACE to Treat Hepatocellular Carcinoma After Operation
NCT05342883	GammaTile and Stupp in Newly Diagnosed GBM
NCT05124002	Recombinant Human Adenovirus Type 5 Plus HAIC of FOLFOX for Intrahepatic Cholangiocarcinoma
NCT05367765	A Real World Study of the Efficacy and Safety of Flumatinib Versus Imatinib in Patients With Newly Diagnosed Chronic Myeloid Leukemia in Chronic Phase
NCT04933526	The Efficacy and Safety of Switching to Flumatinib Versus Dasatinib After Imatinib-related Low-grade Adverse Events in CML-CP Patients
NCT04925141	A Study of Dasatinib as First-line Treatment for Newly Diagnosed Chronic-Phase Chronic Myeloid Leukemia (CML-CP)
NCT04155411	Dose Reduced Dasatinib (70 mg Daily) as First-line Treatment for Newly Diagnosed CML-CP
NCT03332511	Efficacy and Safety of Nilotinib in CML-CP
NCT03216070	Low-dose Dasatinib as First-line Treatment for Chronic Myeloid Leukemia
NCT02602314	Sustained Treatment-free Remission in BCR-ABL+ Chronic Myeloid Leukemia
NCT02317159	Efficacy and Safety of Imatinib Mesylate as First-line Treatment for the Patients With Chronic Phase of Chronic Myeloid Leukemia
NCT04446806	Prevention and Treatment of Differentiation Syndrome in Patients With Acute Promyelocytic Leukemia
NCT02204722	A Study to Evaluate Efficacy and Safety of Glinib in Newly Diagnosed CML Patients
NCT04489888	A Study of Pembrolizumab (MK-3475) Plus Carboplatin and Paclitaxel as First-line Treatment of Recurrent/Metastatic Head and Neck Squamous Cell Carcinoma (MK-3475-B10/KEYNOTE B10)

NCT03388931	Radiotherapy Dose Escalation in Locally Advanced Squamous Cell Carcinoma of the Larynx or Hypopharynx
NCT03196843	Radiotherapy Combine With Raltitrexed Versus Radiotherapy Alone in Older Patients With HNSCC.
NCT04288700	Evaluation of the Efficacy of Captopril Versus Propranolol and Timolol as a Treatment of Infantile Capillary Hemangioma
NCT05191225	Ultrafast Truxima Infusion in Non-Hodgkin's Lymphoma: Txagorapid Study
NCT04726501	CCCG-HD-2018 for Children and Adolescents With Newly Diagnosed Hodgkin Lymphoma
NCT04134247	Study of PD-1 Monoclonal Antibody in Combination With Chemotherapy in Patients With RR NHL
NCT04083066	Open Randomized Prospective Clinical Study of R-FPD Versus R-MAD Regimen in the Treatment of Primary Central Nervous System Lymphoma
NCT03920813	Determinants of Mercaptopurine Toxicity in Paediatric Acute Lymphoblastic Leukemia Maintenance Therapy
NCT03677596	A Study Of Two Inotuzumab Ozogamicin Doses in Relapsed/ Refractory Acute Lymphoblastic Leukemia Transplant Eligible Patients
NCT03500133	Pediatric Hodgkin Lymphoma Treatment Trial With Low Cumulative Doses of Chemotherapy Agents and Reduced Radiation.
NCT02690922	Low-dose Chemotherapy Combine With Tyrosine Kinase Inhibitor to Treat ph+ Acute Lymphoblastic Leukemia Patients
NCT02526823	Clinical Application of Polyethylene Glycol Liposome Doxorubicin (PLD) in Primary Lymphoma
NCT02389920	Multicenter, PhaseⅣ, Open Label Trial of Nilotinib in Adult Patients Diagnosed Philadelphia Chromosome Positive(Ph+) Chronic Myeloid Leukemia in CP/AP Intolerant to Dasatinib
NCT01906671	Study on Two Different Formulations of 6-mercaptopurine. Tablet Versus Oral Liquid
NCT01873807	HD-Idarubicin/Etoposide Intensified Conditioning Regimen Allo-HSCT for Adult ALL
NCT02546674	Study Assessing Deep Molecular Response in Adult Patients With CML in Chronic Phase Treated With Nilotinib Firstline.
NCT05113290	Effect and Safety of Recombinant Human Adenovirus Type 5 in Advanced HCC With Stable Disease After Sorafenib Treatment
NCT04562428	The Safety and Efficacy Evaluation of XSLJZ in Advanced Hepatocellular Carcinoma Patients With Multiple Protein Kinase Inhibitor Therapy: A Randomized, Double-Blind, Controlled Trial
NCT04297254	A Study to Assess the Safety and Efficacy of Lenvatinib as First-line Treatment in Participants With Unresectable HCC
NCT03996681	TACE Combined With Methylcantharidimide Tablets in the Treatment of Large and Unresectable Hepatocellular Carcinoma

NCT03963206	Cabozantinib toLERANCE Study in HepatoCellular Carcinoma (CLERANCE)
NCT04413201	AFAMOSI: Efficacy and Safety of Afatinib Followed by Osimertinib Compared to Osimertinib in Patients With EGFRmutated/T790M Mutation Negative Nonsquamous NSCLC
NCT03007212	Outcome of Transarterial Chemo-embolization (TACE) in Hepatocellular Carcinoma Patients With Partial Portal Vein Thrombosis
NCT02590510	Different Dose of Methotrexate for the Treatment of Meningeal Carcinomatosis
NCT03778853	Study of Anlotinib in Advanced Non-squamous NSCLC Patients in the Elderly Without Systemic Chemotherapy (ALTER-L006)
NCT03768037	Anlotinib Plus Pemetrexed or Pemetrexed for Previously Untreated Elderly (>=70) or PS=2 Non-squamous NSCLC
NCT03755869	Study of Anlotinib in Non-squamous NSCLC Patients Who Failed First-Line（ALTER-L020）
NCT02293356	Phase IV Clinical Trial of Nimotuzumab in the Treatment of Nasopharyngeal Carcinoma
NCT02504983	Clinical Trial for GALNT14 Genotype - Guided, Sorafenib in Combination With TACE in Hepatocellular Carcinoma
NCT02804646	Endostar Durative Transfusion Combined With Chemotherapy in the Treatment of Advanced Lung Adenocarcinoma
NCT02253511	A Prospective Control Study of Cidan Capsule Combined With TACE in Hepatocellular Carcinoma
NCT01997957	A RCT of Oral S-1 in Combination With Sequential HAIC of Oxaliplatin After TACE in Patients With Advanced HCC
NCT02399566	Clinical Trial of Erlotinib and Pemetrexed for Maintenance Treatment in Lung Adenocarcinoma
NCT02961998	Preventive Effect of Celecoxib on Sorafenib-related Hand Foot Syndrome, a Single Center, Randomized Controlled Clinical Trail
NCT03525834	Safety and Efficacy of Everolimus (Afinitor®) in Chinese Adult Patients With Angiomyolipoma Associated With Tuberous Sclerosis Complex.
NCT05235737	The Assessment of Immune Response in Newly Diagnosed Glioblastoma Patients Treated With Pembrolizumab
NCT02386800	CINC424A2X01B Rollover Protocol
NCT03007225	Efficacy and Safety of Drug Eluting Beads TACE in Treatment of HCC in Egyptian Patients
NCT04015245	SNMC (Stronger Neo-Minophagen C) for Acute Hepatitis Post Transarterial Chemoembolization Therapy
NCT04077515	Safety and Efficacy of Low-dose Sirolimus to Kaposiform Hemangioendothelioma
NCT04766827	Albumin-bound Paclitaxel Combined With Cisplatin Versus Docetaxel Combined With Cisplatin Induced Chemotherapy in Advanced Head and Neck Squamous Tummor

NCT04448873	Guided Discontinuation Versus Maintenance Treatment of Sirolimus in Pediatric Patients With Kaposiform Hemangioendothelioma
NCT04921722	Percutaneous Administration of Sirolimus in the Treatment of Superficial Complicated Vascular Anomalies
NCT01898585	An Open-Label Study of Zelboraf (Vemurafenib) in Patients With Braf V600 Mutation Positive Metastatic Melanoma
NCT04042376	A Study of Ibrutinib (PCI-32765) in Chinese Participants With Relapse or Refractory Waldenstrom's Macroglobulinemia (WM)
NCT02844322	The Comparison of RCD Versus BCD in Newly Diagnosed Waldenström Macroglobulinemia
NCT02844309	The Efficacy of TCD Following by TP Maintenance Therapy in Newly Diagnosed WM
NCT05518383	B-cell Mature Non-Hodgkin's Lymphoma Treatment Protocol in Children and Adolescents 2021
NCT05611086	N-Acetylcysteine vs Placebo as Adjunctive Treatment in Pediatric Leukemia
NCT05442554	A Study of Brentuximab Vedotin Treatment in Chinese Adults With CD30-Positive Cutaneous T-Cell Lymphoma
NCT04999878	A Prospective Clinical Study of Ruxolitinib and Etoposide Combined With DDGP Regimen (RUE-DDGP) in Induction Therapy of T/NK Cell Lymphoma-associated Hemophagocytic Syndrome.
NCT04930536	Acalabrutinib Study in Indian Patients With Chronic Lymphocytic Leukaemia & Relapsed and Refractory Mantle Cell Lymphoma
NCT04842318	Rituximab Maintenance Treatment of Newly Diagnosed Follicular Lymphoma After BR or RCHOP or R2: a Multicenter Clinical Study
NCT04490590	A Clinical Trial of Chidamide Combined With Etoposide in Relapsed or Refractory NK/T-cell Lymphoma
NCT04152577	R2 and Combination Chemotherapy Versus R and Combination Chemotherapy in Newly Diagnosed Highly Aggressive B-NHL
NCT04040491	PD-1 Antibody, Chidamide, Lenalidomide and Gemcitabine for Peripheral T-cell Lymphoma
NCT04038411	PD-1 Antibody, Chidamide, Lenalidomide and Etoposide for Relapsed or Refractory NK/T Cell Lymphoma
NCT03817853	An Open-Label, Single Arm Study of Obinutuzumab Short Duration Infusion in Patients With Previously Untreated Advanced Follicular Lymphoma
NCT03707847	Crizotinib Combined With Etoposide Capsule Followed by Auto-HSCT for Relapsed and Refractory ALK+ ALCL
NCT03631862	Treatment of Newly Diagnosed Peripheral T-cell Lymphoma
NCT03579082	A Clinical Trial of Decitabine in Relapse and Refractory Diffuse Large B Cell Lymphoma
NCT03558412	A Clinical Trial of Decitabine in Relapsed or Refractory T-lymphoblastic Lymphoma

NCT03513601	Treatment of Elderly Patients With Diffuse Large B-cell Lymphoma
NCT03376958	Apatinib for Relapsed and Refractory Diffuse Large B Cell Lymphoma
NCT03150602	A Pralatrexate Study in Asian Patients With Peripheral T-cell Lymphoma After Prior Therapy
NCT03071822	Combination Chemotherapy Including Cisplatin, Ifosfamide, Gemcitabine, L-asparaginase, Etoposide and Dexamethasone as Treatment of Newly Diagnosed and Relapsed/Refractory Peripheral T Cell Lymphomas
NCT02992834	Anti-CD19:TCRζ Chimeric Antigen Receptor-T Cells in the Treatment for CD19+ B Cell Lymphoma
NCT05353205	A Study of the Efficacy and Safety of Flumatinib in Patients With Newly Diagnosed Chronic Myeloid Leukemia in Chronic Phase.
NCT02858804	EDOCH Alternating With DHAP for New Diagnosed Younger MCL
NCT02752815	Reduced Chemotherapy in Low Risk DLBCL
NCT02487316	A Study of Treatment ALK(+) Systemic Anaplastic Large Cell Lymphoma With Crizotinib
NCT02323659	Comparison of Methotrexate Versus Interferon-alfa 2b in Patients With Primary Cutaneous T-cell Lymphomas
NCT02228382	Safety And Efficacy Study Of Bosutinib In Patients With Philadelphia Chromosome Positive Chronic Myeloid Leukemia Previously Treated With One Or More Tyrosine Kinase Inhibitors
NCT01909934	Study of Brentuximab Vedotin in Participants With Relapsed or Refractory Systemic Anaplastic Large Cell Lymphoma
NCT05379569	Comparative Study of BFC and BuCy Conditioning Regimen for Allo-PBSCT in Acute B-cell ALL
NCT04790045	Lymph Node Microenvironment Modifications in Patients With CLL Treated With Venetoclax-based Regimens
NCT04677439	Flumatinib in CML-CP Patients With Ph+ Post Imatinib Failure
NCT04320316	A Trial to Assess the Safety and Efficacy of KRN23 in Epidermal Nevus Syndrome (ENS)
NCT02086487	Efficacy and Safety Assessment of NIlotinib in CML Patients With Suboptimal Response on Imatinib Therapy
NCT01605981	Trial Evaluating Nilotinib as Treatment for Newly Diagnosed CML Patients in Accelerated Phase.
NCT03335943	Myelodysplastic Syndrome--CDA-2 Hematological Improvement National Affirmation Study
NCT02013102	A Phase Ⅳ Study of Decitabine in Myelodysplastic Syndrome
NCT05160922	Crizotinib Continuation Clinical Study
